# A new procedure to analyze the effect of air changes in building energy consumption

**DOI:** 10.1186/2052-336X-12-37

**Published:** 2014-01-21

**Authors:** Naira Barkhudaryan, José A Orosa, Gholamreza Roshan

**Affiliations:** 1 Department of Energy and M. P, University of A Coruña, Paseo de Ronda, 51, A Coruña 15011, Spain; 2 Department of Geography, Golestan University, Shahid Beheshti Street 49138-15759, Gorgan, Iran

**Keywords:** Energy, Ventilation, ISO13790, Climate change, Monte Carlo

## Abstract

**Background:**

Today, the International Energy Agency is working under good practice guides that integrate appropriate and cost effective technologies. In this paper a new procedure to define building energy consumption in accordance with the ISO 13790 standard was performed and tested based on real data from a Spanish region.

**Results:**

Results showed that the effect of air changes on building energy consumption can be defined using the Weibull peak function model. Furthermore, the effect of climate change on building energy consumption under several different air changes was nearly nil during the summer season.

**Conclusions:**

The procedure obtained could be the much sought-after solution to the problem stated by researchers in the past and future research works relating to this new methodology could help us define the optimal improvement in real buildings to reduce energy consumption, and its related carbon dioxide emissions, at minimal economical cost.

## Background

Various standards relating to energy use have been set up in the last decade to help mitigate climate change effect on building energy consumption. However, these standards focused mainly on new buildings ignoring, for the most part, existing buildings that will require similar improvement challenges in the near future.

Thus, it becomes urgent for the new standards to develop good practice guides that integrate appropriate and cost effective technologies.

In light of this, different international agencies and research units are attempting to find solutions to this age-old persistent problem, which can be defined as follows: How can optimal building improvements, to lower energy consumption and carbon dioxide emission, be identified? This concept is currently expressed mathematically by researchers as a methodology to define the probability density function of the energy consumption in real buildings. Furthermore, researchers anticipate that once this density function is defined, the next step of interest will be to define better improvement strategies to reduce the energy consumption in buildings towards zero value. Simultaneously, this same procedure will help us define the future effect of the weather on this energy density function distribution for an expected climate change.

To solve this problem, in 2000, several European associations of renewable energy decided to share a common office building to utilize synergies. It was then that the Renewable Energy House concept was born, under the project titled New4old [1]. This concept proved to be very successful. It was from this project that Europe’s capital was provided with a showcase for integration of innovative renewable energy and energy efficiency technologies in a 140 year-old listed building.

Today, Energy Conservation in Buildings and Community Systems (ECBCS) claims that approximately one-third of the primary energy is consumed in non-industrial buildings where it is utilized for space heating and cooling, lighting and appliance operations. In view of this, ECBCS undertakes research and provide an international focus to building energy efficiency. Tasks are performed through a series of annexes that are directed towards energy-saving technologies and activities that support their application in practice [1]. ECBCS conducts a diverse range of activities, both through its individual annexes and through centrally organized development and information exchange. Activities usually take the form of a ‘Task Shared’ Annex in which each country commits to a specified effort level.

Following uncertainties in energy supply and concern over the risk of global warming, many countries have now introduced target values for reducing energy consumption in buildings. In view of this, these international agencies attempt to reduce the energy consumption in buildings from between 15 to 30% in the next few years. To achieve such a target, international cooperation, in which research activities and knowledge can be shared, is seen as an essential factor.

Today, the International Energy Agency [2] is working under the same objective in its different research projects, like the Annex 55 [3] and Annex 56 [4]. Annex 55 is currently being developed. Its scope includes providing decision support data and tools for energy retrofitting measures. These tools will be based on probabilistic methodologies for the prediction of energy use, life cycle cost and functional performance. The ultimate outcome of the project will be to develop knowledge and tools that support the use of probability-based design strategies in retrofitting of buildings to ensure that the anticipated energy benefits can be realized [3].

In continuation of the earlier project, Annex 56 [4] was produced. The project aims at developing rules and procedures, as the basis for future standards, enabling cost effective refurbishment of existing buildings within the international commitments to reduce greenhouse gas emissions (GHG) and for climate change mitigation. This implies rehabilitation towards nearly-zero emission buildings. As in the prior project, one of the main objectives is to provide tools, guidelines, recommendations, best practice examples and background information for policy makers, designers, users, owners and developers that will help to reduce GHG emissions in the existing buildings sector.

All these research projects are being currently developed and, in the initial stage, need to define the more important parameters that influence building energy consumption. In light of this, the ventilation rate was defined as a primary parameter related to indoor air quality (IAQ) and energy saving in earlier years. Furthermore, the effect of ventilation rate on building energy consumption, today and in the near future, under the effect of climate changes, is pending analysis. Consequently, a suitable selection of a ventilation method must be done from among natural, mechanical and hybrid ventilations. In recent research works, based on the tracer gas decay method, these three ventilation methodologies were compared in urban environments to define their performances in accordance with the nominal time constant [5]. These results showed that hybrid ventilation is associated with somewhat higher nominal time constants and higher air exchange efficiency values than natural cross-ventilation and such ventilation efficiencies seem to be higher when the air-exchange rates are lower. The conclusion generally drawn is that residential mixing ventilation and industrial hybrid displacement ventilation are the more significant alternatives for future building design [5]. However, the most favorable number of air changes required for each different ventilation procedure to reduce the energy consumption is still not clear. It is due, most often, to the energy consumption, which is not the main parameter that controls the number of air changes, and it is defined as a function of the type of indoor ambience and activities occurring indoors.

Finally, as mentioned earlier, climate change effect does exert a clear influence on energy consumption. Consequently, recent research studies have begun to analyze the effect of climate change on the building energy consumption due to the ventilation system [6]. In this study, measurements of temperature and relative humidity were performed in office buildings for extended periods of time, which allow us to predict the energy consumption for the next few years, under new temperatures, due to climate change. This was done based on the general equation of energy for building certification, in accordance with EN ISO 13790 [7,8] and related ASHRAE standards [9]. From the results the initial conclusion drawn was that winter ventilation experiments showed a higher utilization factor and that this was implemented with the increase in air changes. Despite this, lower energy consumption was observed in the winter than in the summer, and it was noted that when air changes increased, this effect revealed the opposite behaviour. Therefore, it was concluded that it will be of interest to increase the air changes during the summer season for the next 10 years.

After this review it can be concluded that economically optimized renovation strategies for different local conditions and categories of existing buildings are still to be developed and demonstrated. In view of this, the goal of this present research work is to show one possible solution to this problem, based on the ISO 13790 standard and different stochastic procedures.

## Methods

In the initial stage, real sampled data from Galicia (Spain) buildings was employed to define the input data of ISO 13790 standard and its upper and lower working values. Parameters like indoor air temperature, number of air changes and energy consumption were sampled in a real building and, at the same time, it were employed for the energy consumption calculation of the same building in accordance with the ISO 13790 standard. As a result, the calculation procedure was validated. Finally, in accordance with Monte Carlo method, this building energy consumption was calculated in accordance with ISO 13790 standard employing random input values of each sampled variables and showing, after an adequate curve fitting, the probabilistic model of the building.

### Real sampled data

#### Weather conditions

**Figure 1:**
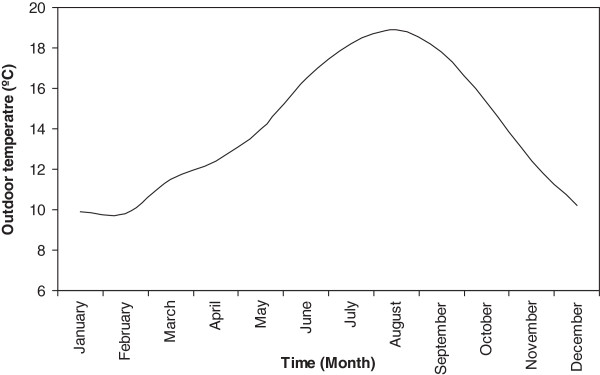
Weather conditions.

In the initial stage, the local weather conditions were defined in accordance with the nearest weather stations [10]. Results are shown in Figure 1.

### Climate change models

Climate change models were obtained from earlier research works on the weather conditions in the local region, which was the objective of this study. In this study, after a curve fit for each season, different models were obtained in accordance with the Equations (1), (2), (3) and (4). Each equation revealed the average temperature rise in Galicia for the last 30 years, showing the reference value of **13.63**°C in 1987. This same study showed a relationship between climate change and a slight reduction in the Galician summer north wind frequencies.

(1) $$\Delta t_{w\mathrm{int}\mathrm{er}}=0.05\cdot \tau$$

(2) $$\Delta t_{\mathrm{spring}}=0.07\cdot \tau$$

(3) $$\Delta t_{\mathrm{Summer}}=0.05\cdot \tau$$

(4) $$\Delta t_{\mathrm{Autumn}}=0.03\cdot \tau$$

where *Δt* is the mean seasonal temperature increase (°C) and *τ* is the time (years).

**Figure 2:**
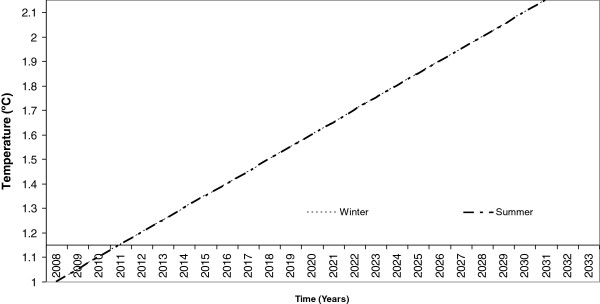
Climate change model.

Once these models are obtained and assuming a linear temperature increase with time, they can be used to predict the climate change in Galicia for the next 20 years, as seen in Figure 2.

### Monte Carlo method

For this case study, the Monte Carlo method was applied by Microsoft Excel and implemented with Visual Basic for Applications of the ISO 13790 standard calculation procedure. This methodology permits us to easily define algorithms within a reduced time period.

### Weibull model and curve fitting

**Figure 3:**
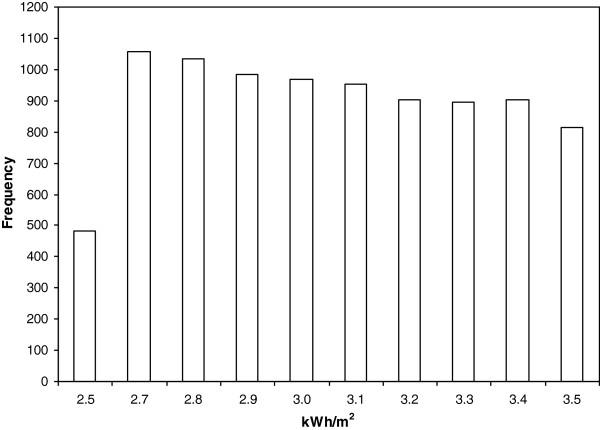
Histogram.

Different curve fitting processes were developed with the data obtained from each group of runs once the histogram was defined, as seen in Figure 3. Consequently, different probabilistic density functions were evaluated to define this data obtained. For example, Normal, Log Normal and Weibull distribution were proposed among others.

## Results and discussion

As the primary results, different charts showing the random energy consumption and carbon dioxide emission were represented in Figures 4, 5, 6 and 7. Furthermore, different Weibull models for the heating and cooling seasons under different air changes were represented in Figures 8, 9, 10, 11, 12, 13, 14 and 15.

**Figure 4:**
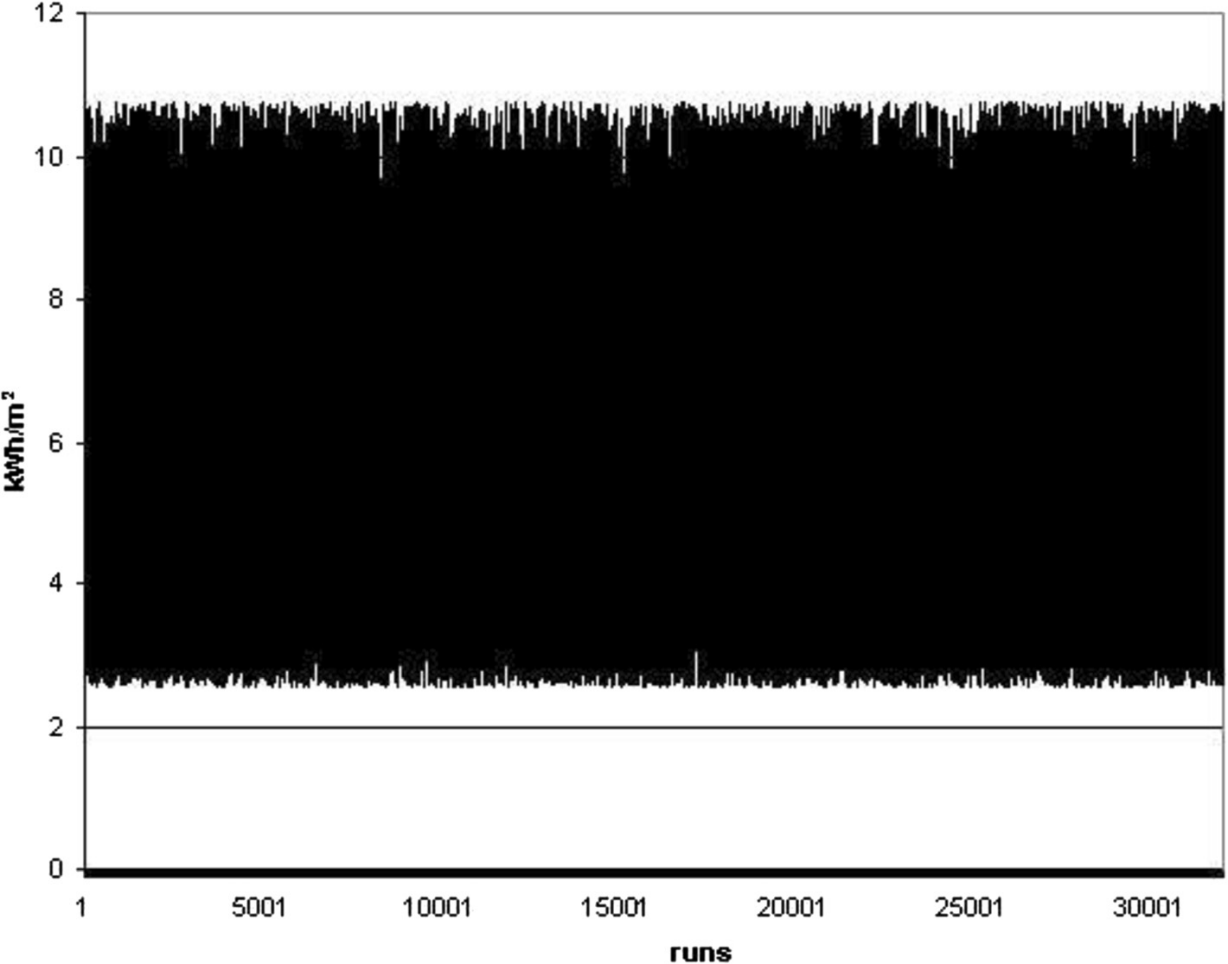
Cooling energy.

**Figure 5:**
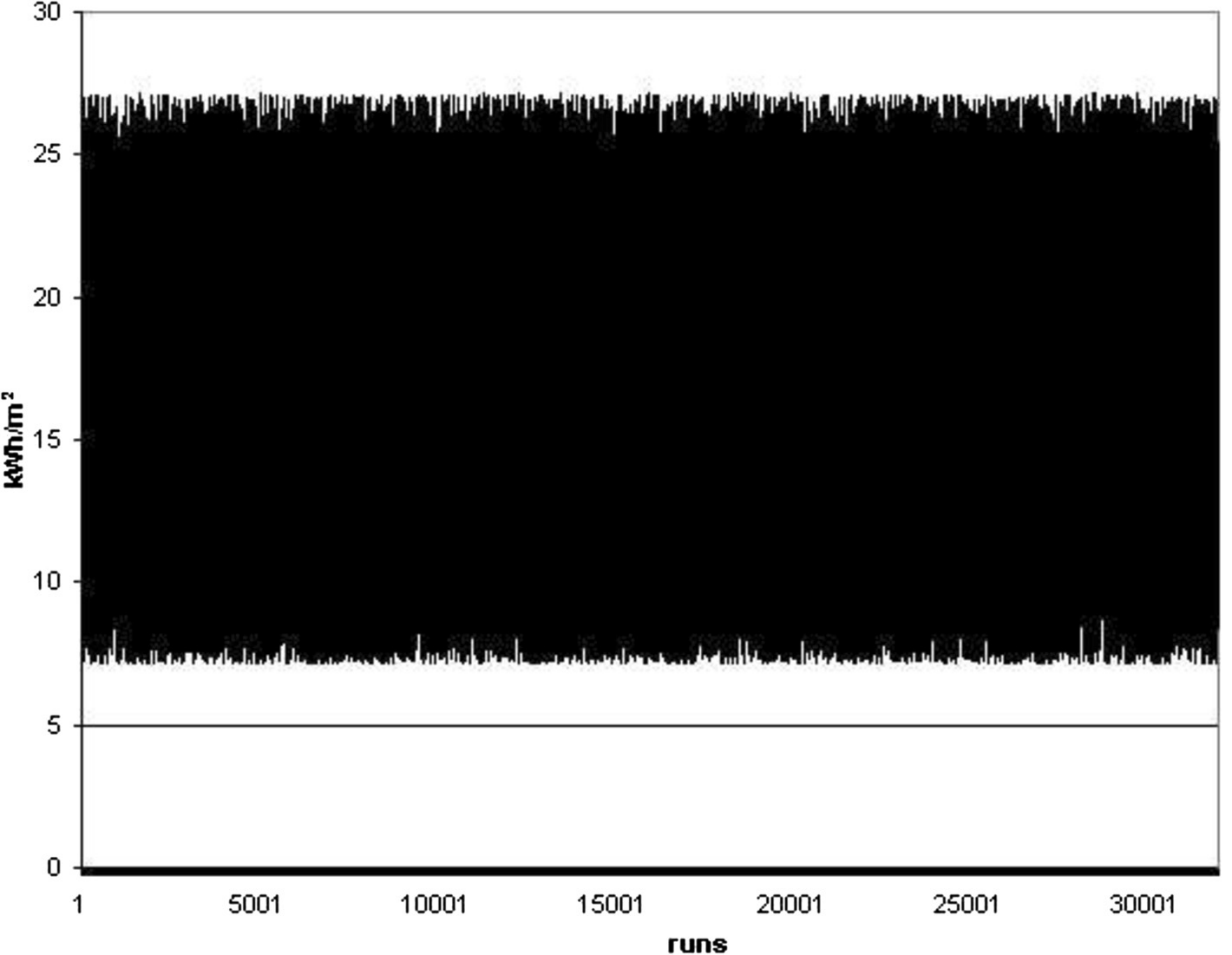
Heating energy.

**Figure 6:**
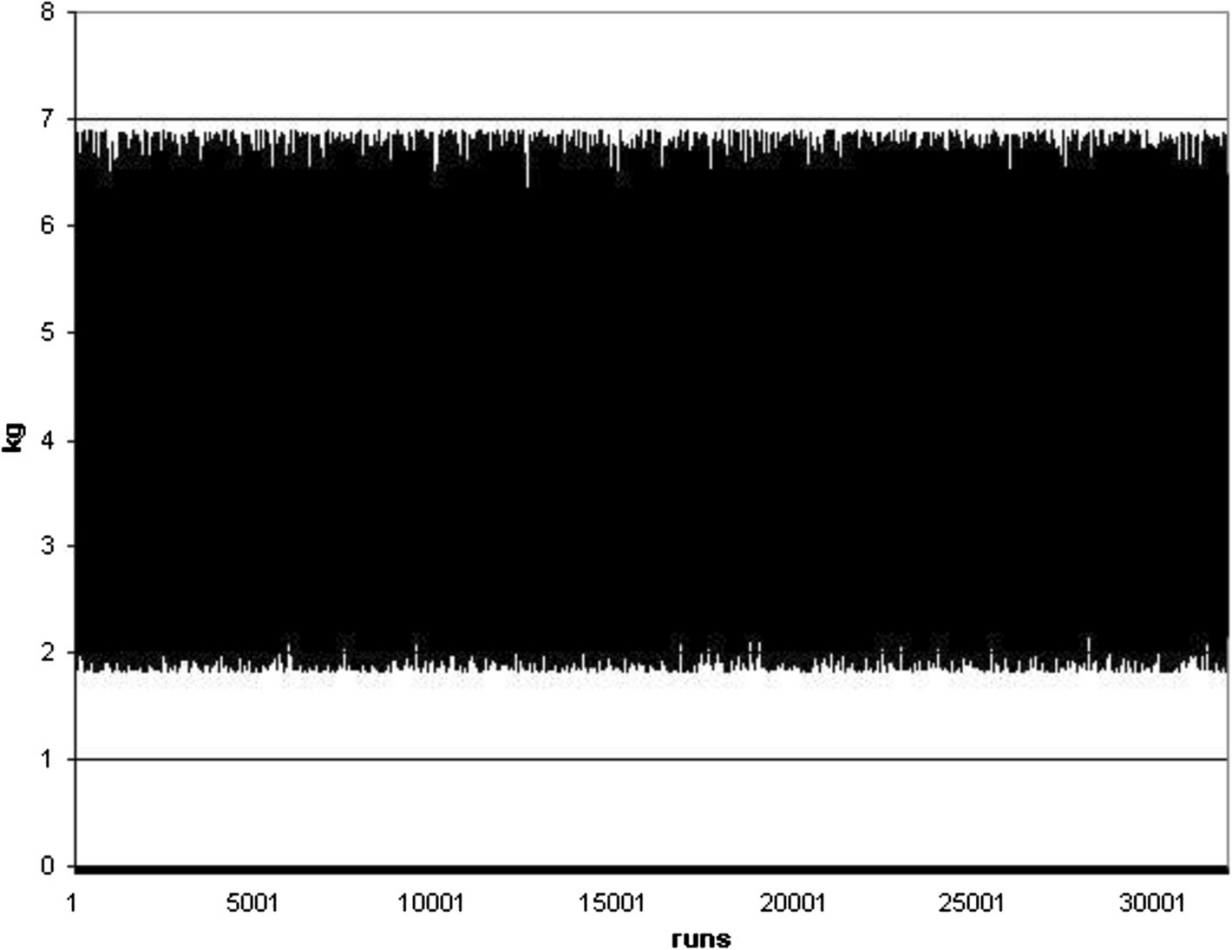
**CO**_**2 **_**emission due to cooling energy.**

**Figure 7:**
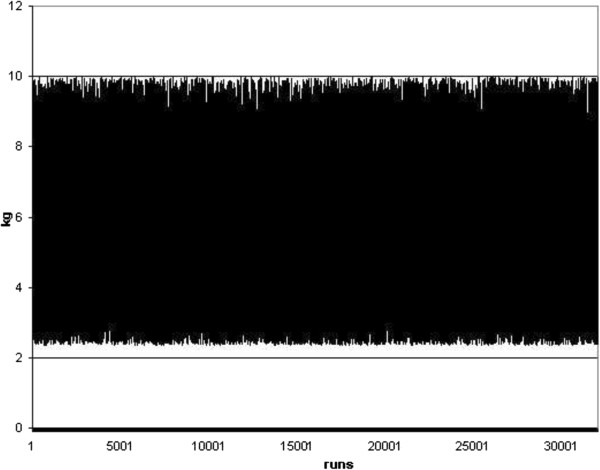
**CO**_**2 **_**emission due to heating energy.**

**Figure 8:**
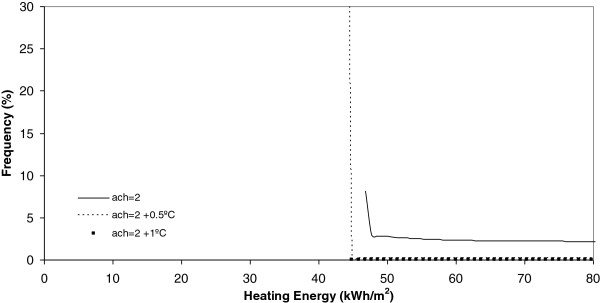
Heating energy under 2 ach.

**Figure 9:**
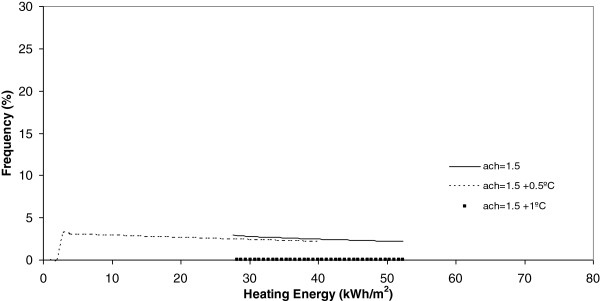
Heating energy under 1.5 ach.

**Figure 10:**
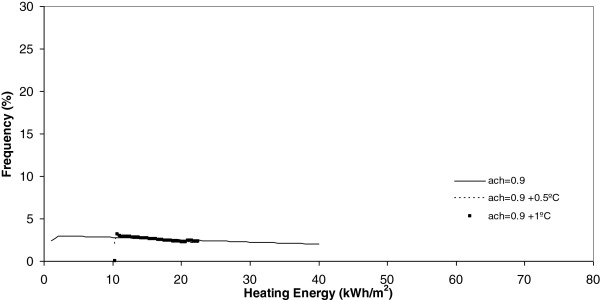
Heating energy under 0.9 ach.

**Figure 11:**
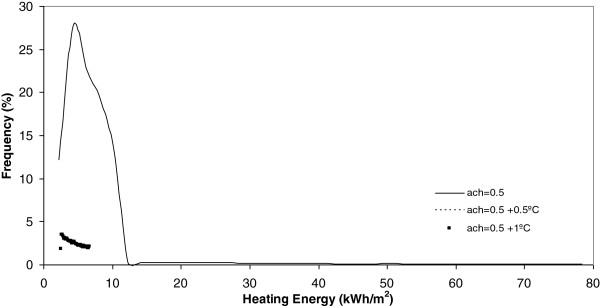
Heating energy under 0.5 ach.

**Figure 12:**
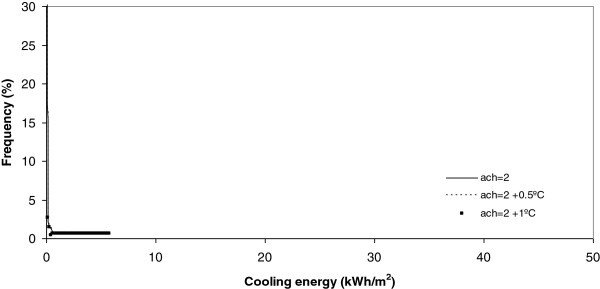
Cooling energy under 2 ach.

**Figure 13:**
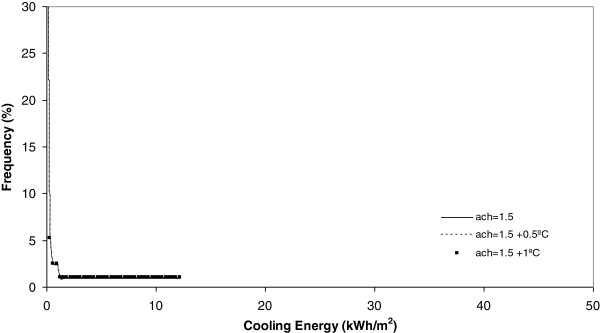
Cooling energy under 1.5 ach.

**Figure 14:**
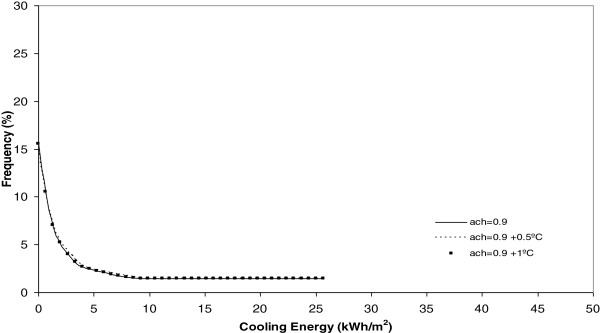
Cooling energy under 0.9 ach.

**Figure 15:**
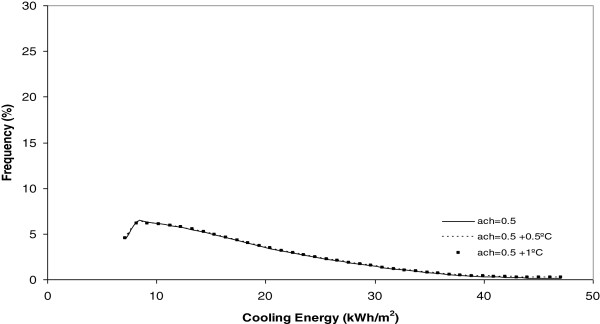
Cooling energy under 0.5 ach.

**Figure 16:**
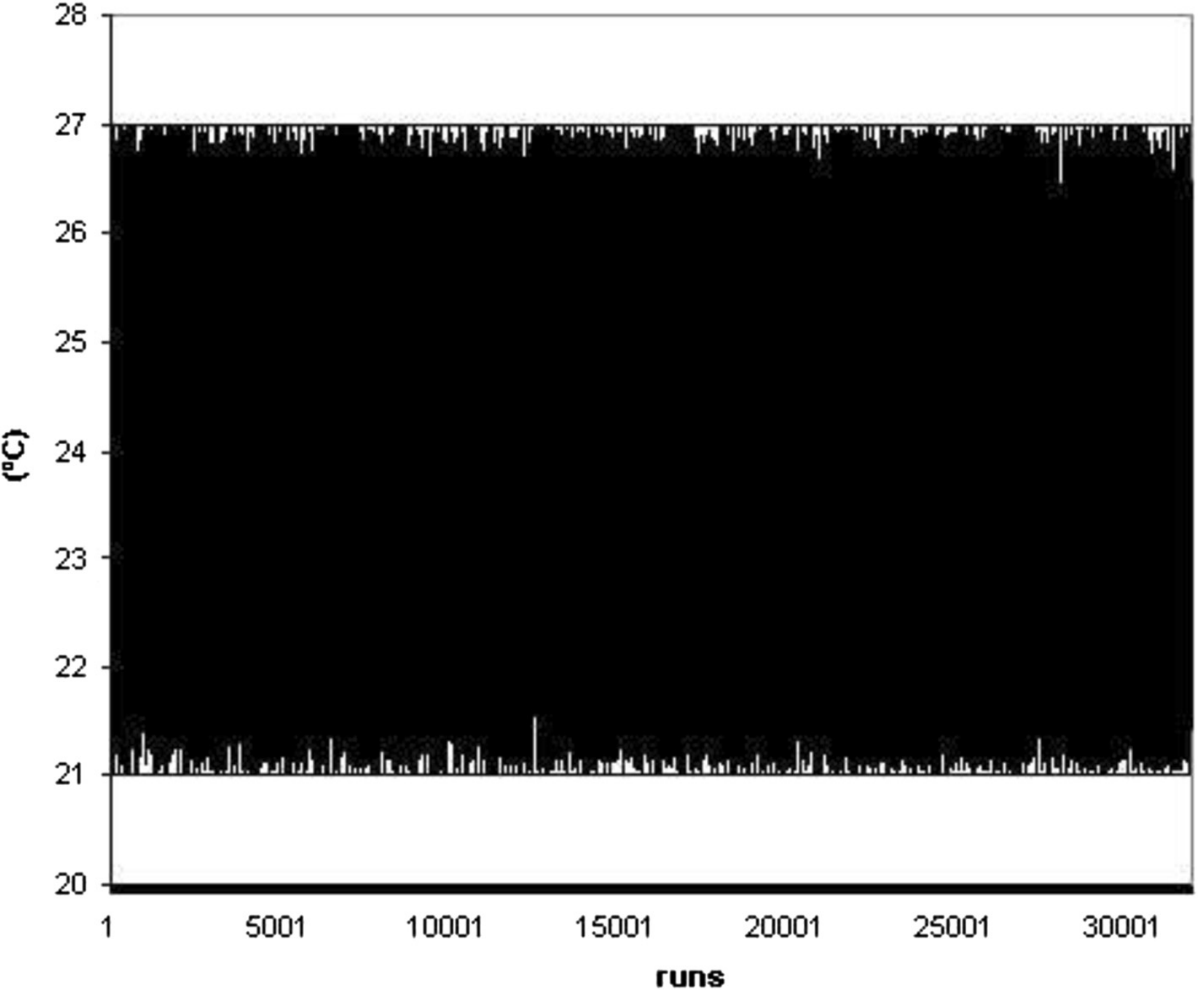
Summer indoor temperature proposed randomly.

**Figure 17:**
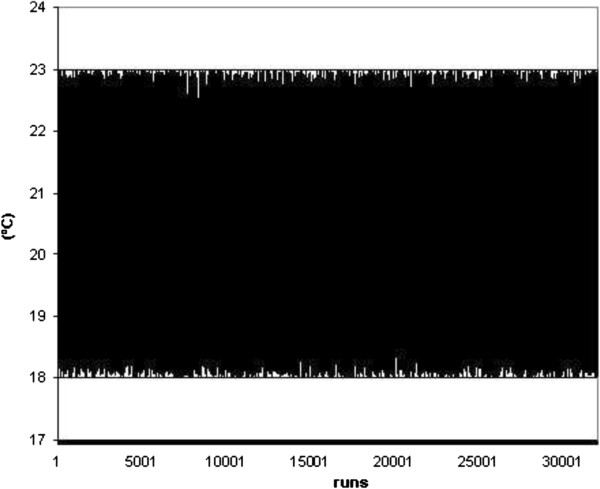
Winter indoor temperature proposed randomly.

As seen in Figures 16 and 17, the main parameters employed randomly into the ISO 13790 standard are represented. Particularly, indoor air temperature was defined in the range from 23°C to 27°C during the summer and from 18°C to 21°C during the winter season. During this simulation process, other parameters were considered constant in accordance with the data sampled in real buildings. For example, the number of occupants was fixed as five persons, as this is considered the mean occupation value in this office building. In accordance with building construction materials, the thermal inertia was defined as low and equal to 150 kJ/m^2^K. Finally, the building used for this study is a basement of size 120 m^2^.

Once the mean indoor parameters of a real building were defined, then the weather conditions surrounding the building used in this study were defined. Significantly, the building is located in the north-west of Spain and presents a mild and humid climate, as seen in Figure 1. However, as mentioned earlier, a climate change, for this region, in the next 10 and 20 years is expected, with a mean increment of 0.5°C every ten years, as seen in Figure 2, in keeping with the earlier research works.

Once the input data was defined, the Monte Carlo method could be employed. Calculation was performed in accordance with ISO 13790 indications, and parameters such as the heating and cooling system efficiencies were fixed at 0.7 and 2.55, respectively. At the same time, the carbon dioxide emission was defined as a function of the energy consumption obtained for each season. Finally, for this particular case study 50,000 runs were applied, in keeping with the convergence criterion.

Prior to analyzing the energy consumption in buildings, it must be clarified that the ISO 13790 standard considers different kinds of energy consumption. The first one is due to the energy required to ventilate under different numbers of air changes, whereas the other energy consumption is related to the heating or cooling system to reach adequate indoor conditions. Consequently, the present study analyzes the effect on the cooling and heating energy consumption, under different number of air changes.

Results of Figures 4 and 5 showed the energy consumption during the heating and cooling seasons, once each random run was completed on this standard. Simultaneously, the carbon dioxide emission related to this energy consumption was represented in Figures 6 and 7. From these Figures it can be concluded that the energy consumption in this building during the cooling season must be between 3 and 11 kWh/m^2^ and that during the heating season it changes to between 7 and 27 kWh/m^2^. This result clearly concurs with real sampled data and the information obtained on the energy consumption during these two seasons. At the same time, the carbon dioxide emissions defined in Figures 6 and 7 show an emission range between 2 and 7 kg, as well as 3 and 10 kg of CO_2_ during the summer and winter season, respectively.

Once each different group of runs was completed, the results were grouped in accordance with their frequency, as shown in the histogram of Figure 3. After this, a curve fit process was done and the results showed the better common curve fit model for each group of runs. In particular, the Weibull peak function model was selected as the only one suitable to be employed with an adequate correlation factor, for all curves obtained. In view of this, the correlation factor reached 0.9 in most cases and, consequently, the curve fit was considered adequate. This result is of particular interest for researchers, as these models, and the previously obtained thermal inertia [11], can be easily optimized by different control systems reducing the energy consumption and being the initial tool for an optimal building design improvement.

If each different chart is analyzed, it is evident, for example Figure 8, that today, the heating energy consumption is higher when the number of air changes is maximum and equal to 2 ach. Furthermore, in this same Figure it is seen that when the climate change effect increases outdoor temperature by 0.5°C or 1°C, this energy consumption, under the same number of air changes experiences a clear decrease towards zero.

Another conclusion obtained from Figures 9 and 10 is that, when the number of air changes is reduced with values of 1.5, 0.9 and 0.5, the model of energy consumption experiences s a clear movement towards the left. This movement implies a reduction in the energy consumption. For example, in these Figures it is evident that the energy consumption obtained today is the higher one and that, the higher the climate change effect, the more reduced is the energy consumption during the winter season. This conclusion is clearly in accordance with common sense and is seen in Figure 11. In this Figure, below the minimum number of air changes of 0.5, there are a higher number of states with energy consumption below 10 kWh/m^2^.

On analysis the effect of the number of air changes over the summer cooling energy consumption as Figures 12, 13, 14 and 15 show indicates there is no influence on the outdoor temperature and the energy consumption obtained today, or in the next ten and twenty years. However, it is seen that the higher the number of air changes, the lower the energy consumption, in this season. An extreme case is obtained when we define the number of air changes as 0.5, as shown in Figure 15.

Concurring with the results obtained from the earlier works, it was concluded that during winter, there is lower energy consumption than in the summer. Despite this, when air changes are increased, this effect experiences a clear change, revealing the opposite behavior. To summarize, it can be concluded that the global energy consumption in the next few years will be constant during the winter and that, during the summer, a decrease will be offered if the air changes increase to 1.5 or more. As a result, it can be concluded that this will be of great interest to future designers. From the energetic point of view it is interesting to increase the air changes during summer season for the next 10 years. This increase in air changes can be reached by mechanical ventilation or various other methods like infiltration. Despite this, as it is well known, the ventilation rate must consider other parameters like indoor activities and pollutants [12,13] and not energetic point of view alone to define the optimal ventilation rate.

## Conclusions

In the present research work, a new stochastic procedure to define the effect of the ventilation rate and climate change on a real building energy consumption and carbon dioxide emission was performed. Results showed the energy consumption during the heating and cooling season in a real building, once each random run was completed based on the ISO 13790 standard. It was concluded that the energy consumption during the cooling season must be between 3 and 11 kWh/m^2^ and that during the heating season it changed to between 7 and 27 kWh/m^2^. This result is in clear agreement with real sampled data and the information obtained on the energy consumption during these two seasons. At the same time, carbon dioxide emissions were defined as showing an emission range between 2 and 7 kg, and 3 and 10 kg of CO_2_ during the summer and winter season, respectively.

Once each different run was completed, the results were grouped in keeping with its frequency and, after this, a curve fitting was done and the results showed that a better common curve fit for each run was obtained using the Weibull peak function model. At the same time, the heating energy consumption was recognized to be higher today under a higher number of air changes (2 ach). Furthermore, when climate change effects increase outdoor temperature by 0.5°C or 1°C, this energy consumption, under the same number of air changes, experiences a clear decrease towards zero. When the number of air changes is reduced, with values of 1.5, 0.9 and 0.5, the model of energy consumption experiences a clear movement towards the left. The energy consumption currently obtained is the higher one and, the higher the climate change effect, the more reduced the energy consumption during the winter season.

On analyzing the effect of the number of air changes on the summer cooling energy consumption it was obtained that, currently, there is no difference between the effect of temperature and energy consumption for the next ten and twenty years. However, it is evident that the higher the number of air changes the lower the energy consumption in this season. This effect is clear under a number of air changes of 0.5.

Concurring with the results obtained from the earlier works, it was concluded that, during winter, there is a lower energy consumption than in the summer and that, when the air changes increase, this effect experiences a change achieving the opposite behavior. Therefore, it can be concluded that this will be of significant interest to future designers. From the energetic point of view it is interesting to increase the air changes during the summer season for the next ten years.

Finally, the procedure obtained could be the much sought-after solution to the problem stated by researchers in the past and future research works relating to this new methodology could help us define the optimal improvement in real buildings to reduce energy consumption, and its related carbon dioxide emissions, at minimal economical cost.

## Competing interest

The authors do not have any competing interest.

## Authors’ contributions

NB carried out the numerical analysis, conceived of the study, and participated in its design and coordination and helped to draft the manuscript. JO carried out the curve fitting. GR carried out the ISO 13790 implementation. All authors read and approved the final manuscript.
